# Perspective: causes and functional significance of temporal variations in attention control

**DOI:** 10.3389/fnhum.2013.00381

**Published:** 2013-07-23

**Authors:** Agatha Lenartowicz, Gregory V. Simpson, Mark S. Cohen

**Affiliations:** ^1^Department of Psychiatry and Biobehavioral Sciences, Semel Institute for Neuroscience and Human Behavior, University of California Los AngelesLos Angeles, CA, USA; ^2^Attention Research InstituteSan Francisco, CA, USA

**Keywords:** attention control, fluctuations, network interactions, attention deficits, internal cognition

## Abstract

Attention control describes the human ability to selectively modulate the plethora of sensory signals and internal thoughts. The neural systems of attention control have been studied extensively, warranted by the importance of this ability to daily functioning. Here, we consider an emerging theme in the study of attention control—slow temporal fluctuations. We posit that these fluctuations are functionally significant, and may reflect underlying interactions between the neural systems related to attention control. We explore thought experiments to generate different perspectives on landscapes created by the interactions between attention control networks and the sources of input to these control systems. We examine interactions of the fronto-parietal and the default mode networks in the context of internal cognition, and the noradrenergic modulatory projections in the context of arousal, and we consider the implications of these inter-network dynamics on attention states and attention disorders. Through these thought experiments we highlight the breadth of potential knowledge to be gained from the study of slow fluctuations in attention control.

## Attention control and fluctuations

**Figure 1:**
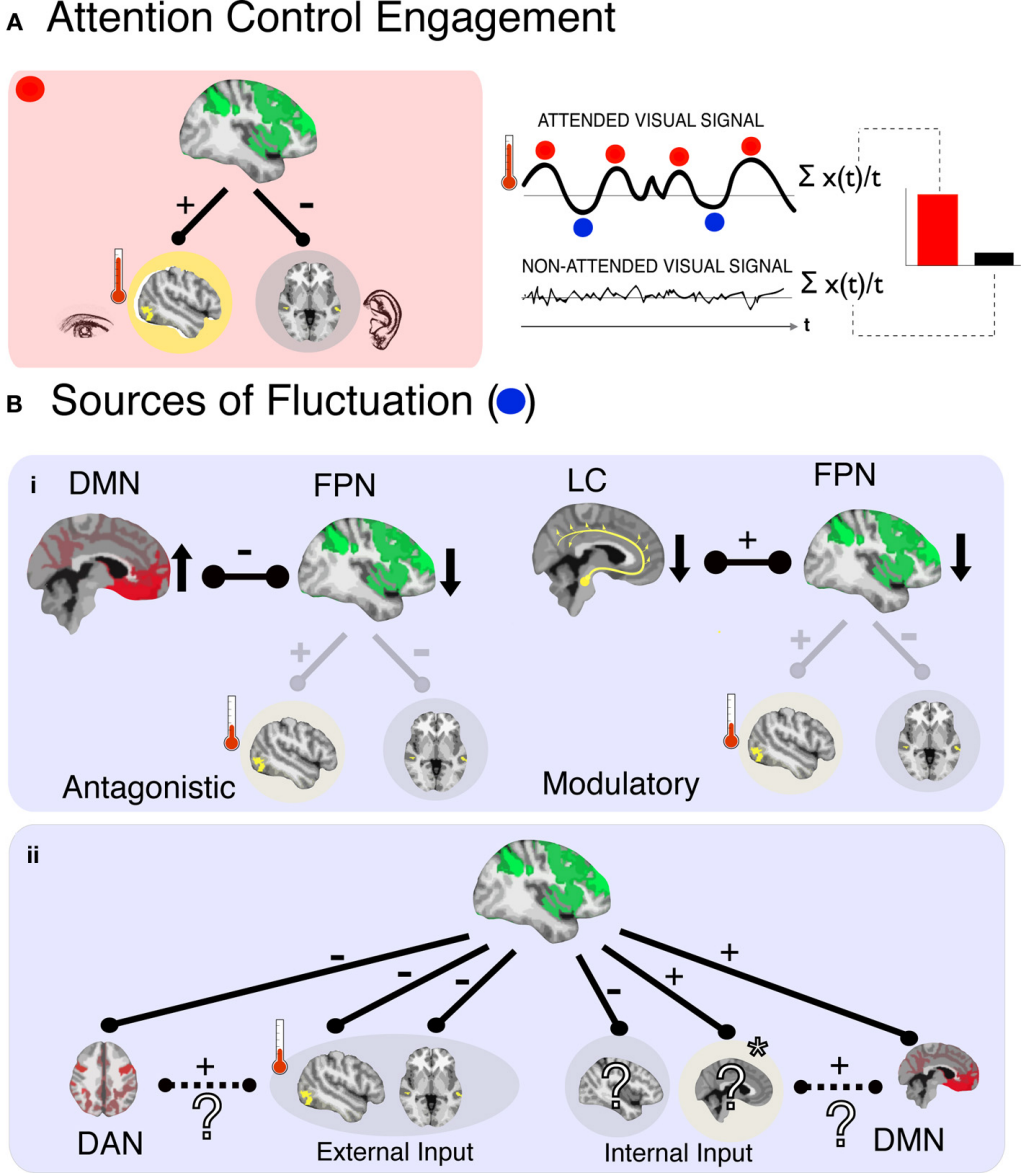
**In a representative scenario of attention control and its fluctuations (A), (left panel) activity corresponding to an attended signal (e.g., visual cortex) is enhanced (red thermometer level is high) and activity corresponding to an ignored signal (e.g., auditory) is suppressed, due to control signals from the FPN.** The magnitude of enhancement is quantified by comparing activity for the target stimulus when it is attended relative to when it is ignored (right panel). This can be conceived as an average index of “moment-to-moment” attention, an approach that ignores any underlying fluctuations in the attended signal amplitude (blue and red dots) that may be related to functionally significant variations in attention control. Potential sources of such fluctuations are shown below **(B)**, and can be system-based (i) and input-based (ii). In the case of systemic sources a decrease in FPN activity could arise by (i), the antagonistic influence of another network (left; DMN–default mode network) or decreased modulatory input (right; LC–locus coeruleus noradrenergic inputs) that decrease activity in FPN, leading to an attenuation of control over sensory processing regions, and therefore lower indices of control as measured in the target processing region (red thermometer level is low). In the case of input-based fluctuations, FPN activity level does not change but is redirected to a different processing input (ii). In this example attention is oriented toward an internal input (e.g., a memory, indicated by ^*^), resulting in a decrease in responses to other inputs—including other internal inputs (e.g., planning dinner) and the external target (visual) inputs. The identity of cortical regions that process internal inputs is unknown (?), as are the interactions of such regions with higher-order networks (e.g., DAN/DMN). We posit here one possibility, that the input cortices and higher-order networks responsible for their processing will be positively correlated in their relationship with FPN. In the example here, the involuntary capture of attention by an internal input leads to positive correlations between DMN and FPN.

Attention control allows us to ignore distracting information so that we may focus selectively on information relevant to goal directed behavior. Copious research has demonstrated that attention control involves “top-down” signals from association cortices, biasing activity in sensory regions to enhance the magnitude of attended signals; and evidence is building to show that top-down processes also suppress the magnitude of ignored signals (Posner and Dehane, 1994; Desimone and Duncan, 1995; Miller and Cohen, 2001). Integral to top-down biasing is the fronto-parietal network (FPN, also referred to as the “executive control network”) (Corbetta and Shulman, 2002; Dosenbach et al., 2006; Fox et al., 2006; Raichle, 2011), a network encompassing dorsal and medial prefrontal cortices and superior parietal cortices, that acts to distinguish attended from ignored signals (Ruff and Driver, 2006; Gazzaley et al., 2007; Capotosto et al., 2009) (Figure 1A, *left panel*). Recruitment of this system is thought to occur when multiple sensory signals compete for processing resources (Braver and Cohen, 2000; Botvinick et al., 2001; Miller and Cohen, 2001) and/or at the trigger of a salient orienting signal (e.g., novel or loud sound, such as a fire alarm; Posner and Petersen, 1990; Posner and Dehane, 1994).

Attention control also appears to be a fluctuating system. Castellanos et al. (2005) showed that the speed of response in an attention task increases and decreases over a period of 15 s (0.068 Hz) or so, and that these fluctuations are particularly pronounced in children with attention deficit hyperactivity disorder (ADHD). Monto et al. (2008) showed that the accuracy in a simple detection task fluctuates in runs of 10–100 s, that track electrophysiological fluctuations of the same period. The activity of the FPN also shows fluctuations in this frequency range (Vincent et al., 2008). These findings indicate that attention control integrity varies across time, and that this variability has implications for behavior and disease. In contrast, predominant models of attention are concerned with what we term “moment-to-moment attention” (Figure 1A, *right panel*); they explain how attention control influences the processing of discrete, primarily external, sensory events by averaging attention signals across moments—and as such across fluctuations—in time. In the current perspective we therefore explore the emerging question, *what are the causes and functional significance of temporal variations in attention control*?

## Systemic and input sources of fluctuations

If we consider the FPN as a core system that underlies attention control, then fluctuations of attention control (proportional to the strength of modulation of the relative strengths of target and distractor signals, Figure 1A) likely indicate fluctuations in the efficacy of this system. Sources of these fluctuations may be classified further as either *systemic* or *input*. A systemically-based fluctuation in efficacy would be defined as a limitation in FPN functionality occurring when the entire system is temporarily less active, either because of operational characteristics (e.g., the entire system is activated insufficiently) or because of negative interactions with other neural systems (e.g., its activity is suppressed by another system)—with no change to the inputs. An input-based fluctuation would be defined as a misdirection of FPN activity relative to a desired goal, such as when attention control is rerouted by distracting signals, be they external signals or, as we consider here, internal thoughts—without a change in FPN activation. Therefore, a key to understanding the fluctuations in efficacy of attention control is knowledge of the conditions for, and products of, the interactions of FPN with other neural systems and inputs. We consider here two such candidates, arousal and internal cognition.

### The case of arousal

The idea of a systemic fluctuation of FPN is demonstrated readily in the effect that chemical neuromodulators have on its efficacy. All four of the primary neuromodulators, the catecholamines (dopamine and noradrenaline), acetylcholine and serotonin, have been shown to affect attention (Foote and Morrison, 1987; Coull, 1998; Briand et al., 2007; Rokem et al., 2010). For brevity we focus on the example of the noradrenergic (NE) system (for a comprehensive review see Moore and Bloom, 1979; Foote et al., 1983; Berridge and Waterhouse, 2003), often referred to as the LC-NE system because all of its cortical projections arise from a single nucleus in the brainstem, the locus coeruleus (LC). The LC-NE system is of interest because, being part of the reticular activating system (Moruzzi and Magoun, 1949), historically it has been associated with arousal (Berridge and Waterhouse, 2003). In turn, arousal is a prerequisite for attention. Indeed decreased firing of LC neurons is correlated both with drowsiness (Roussel et al., 1967; Aston-Jones and Bloom, 1981) and with poor attentional performance (Mason and Iversen, 1978; Aston-Jones et al., 1999). Excess LC firing, like excess arousal, is also detrimental to performance (Aston-Jones et al., 1999).

How do these observations contribute to systemic fluctuations of FPN? The LC-NE system has diffuse projections across cortex, with terminals that include the FPN (Moore and Bloom, 1979). The effect of NE, specifically, is to decrease spontaneous firing and increase the evoked response (Foote et al., 1975), interpreted as an increase in fidelity and gain of the neuronal response (Aston-Jones and Cohen, 2005). The LC-NE system therefore influences the responsivity of FPN to inputs (as well as of other systems) when attention control is required. This suggests that the LC-NE system could contribute to fluctuations of attention control when the LC-NE system is either under- or overactive, translating into a weaker or stronger response of the FPN as an entire system, given no change in the inputs (Aston-Jones and Cohen, 2005; for a complementary interpretation see Corbetta and Shulman, 2002; Corbetta et al., 2008). This is therefore a *systemic* not an *input* fluctuation of attention control. A weaker response of the FPN would translate into weaker modulatory control over target regions, meaning less target enhancement and less distractor suppression (Figure 1B-i, *right panel*).

### The case of internal cognition

A very different example of a seemingly systemic fluctuation is internal cognition, which refers to thinking; it encompasses mind wandering, self-evaluation, problem solving and active remembering (Giambra, 1995; Smallwood and Schooler, 2006; McVay and Kane, 2009, 2010; Schooler et al., 2011; Christoff, 2012; Smallwood, 2012), processes that have been associated with the activation of a group of functionally connected regions that include medial prefrontal cortex, posterior cingulate cortex, restrosplenial cortex, as well as medial temporal and lateral inferior parietal cortices (Binder et al., 1999; Gusnard et al., 2001; Johnson et al., 2002; Gordon et al., 2007; Mason et al., 2007; Buckner et al., 2008; Christoff et al., 2009; Andrews-Hanna et al., 2010; Stawarczyk et al., 2011). Together these regions comprise the so-called default mode network (DMN) (Shulman et al., 1997; Mazoyer et al., 2001; Raichle et al., 2001; Buckner et al., 2008).

Internal cognition is of particular interest because thinking can, in principle, interfere with attention control over external inputs both through systemic and input pathways. As a systemic influence thinking can be considered a competitor to FPN activity (Figure 1B-i, *left panel*). This hypothesis is supported by early findings showing that FPN activity is correlated negatively with that of the DMN (Fox et al., 2005; Fox and Raichle, 2007). In turn, DMN activity is correlated positively with lapses of external attention (Weissman et al., 2006), reflecting moments when participants are off-task (Buckner et al., 2008; Andrews-Hanna, 2012) and have decreased control over external signals (Weissman et al., 2009; Schooler et al., 2011; Smallwood et al., 2012). Accordingly, fluctuations of attention control could be interpreted as instances during which DMN activation suppresses activity in the FPN and attention control is disrupted by internal cognition, a systemic fluctuation of attention in reference to external signals.

A model based on antagonistic interaction between FPN and DMN, while a useful starting point, is likely an oversimplification of the underlying dynamics. It assumes that internal control and external control are independent, antagonistic systems, which leads to the difficult question: “*Who*” determines which type of control is “on” at any given time? Plausibly, the FPN and DMN interact within a negative feedback circuit (where each suppresses the other), and their individual engagement is determined by the strength of their relative inputs. This does not seem consistent with our ability to quickly switch between internal and external cognition—with no change in inputs. Alternatively, some other system determines whether external attention control or internal cognition is engaged (Sridharan et al., 2008; Leech et al., 2012). A more parsimonious interpretation is that FPN *is* that other system. Namely, DMN activity can be thought of as another input into FPN that is suppressed when attention is oriented externally—resulting in an apparent negative correlation between the two systems. The activation of DMN while attempting to attend to external signals would then be thought of as an input-based source of fluctuation (Figure 1B-ii).

Perhaps most telling with regard to this notion is the observation that in some circumstances the DMN and FPN are correlated positively (Christoff et al., 2009; Spreng et al., 2010; Smallwood et al., 2012; Spreng et al., 2013), arguing against a strictly antagonistic relationship. In these studies, the authors proposed that the positive coupling between FPN and DMN was interpreted more appropriately as attention control working in the service of internal cognition. For instance Spreng et al. (2010, 2013) reported a positive correlation between FPN and DMN during retrieval of autobiographical memories, but not when participants engaged in a visuospatial task. This may be interpreted as attention control being oriented toward memory retrieval, biasing which internal signals (corresponding to memories) were to be retrieved and which were to be ignored.

These observations are consistent with the existence of a single attention control system, the FPN, which can be oriented to process internal or external sources of input (Figure 1B-ii). Moreover, these sources of input may correspond to other potential control systems – such as the DMN. Coincidently, during a visuospatial task, Spreng et al. (2010, 2013) found that the FPN was no longer coupled with the DMN, but was instead correlated positively with activities in frontal eye fields and inferior parietal sulci, which comprise the dorsal attention network (DAN), a specialized control system involved in visuospatial attention. From this perspective, internal cognition can lead to fluctuations of attention control by reorienting FPN away from systems controlling external inputs (e.g., interaction with DAN to process visuospatial information) and toward systems controlling internal inputs (e.g., interaction with DMN in the service of memory retrieval). One hypothesis that arises here is that such orienting of the FPN would be expected to suppress external signals in general (along with inappropriate internal signals such as false memories in the autobiographical retrieval example). Direct support for this idea has been reported: Mind wandering—a well-known example of an attention lapse—is associated with decreased processing of *both* attended and ignored external signals (Weissman et al., 2006; Smallwood et al., 2008; Barron et al., 2011; Kam et al., 2011), a phenomenon referred to recently as “perceptual decoupling” (Smallwood et al., 2007; Schooler et al., 2011).

## A landscape of attention control and outstanding questions

**Figure 2:**
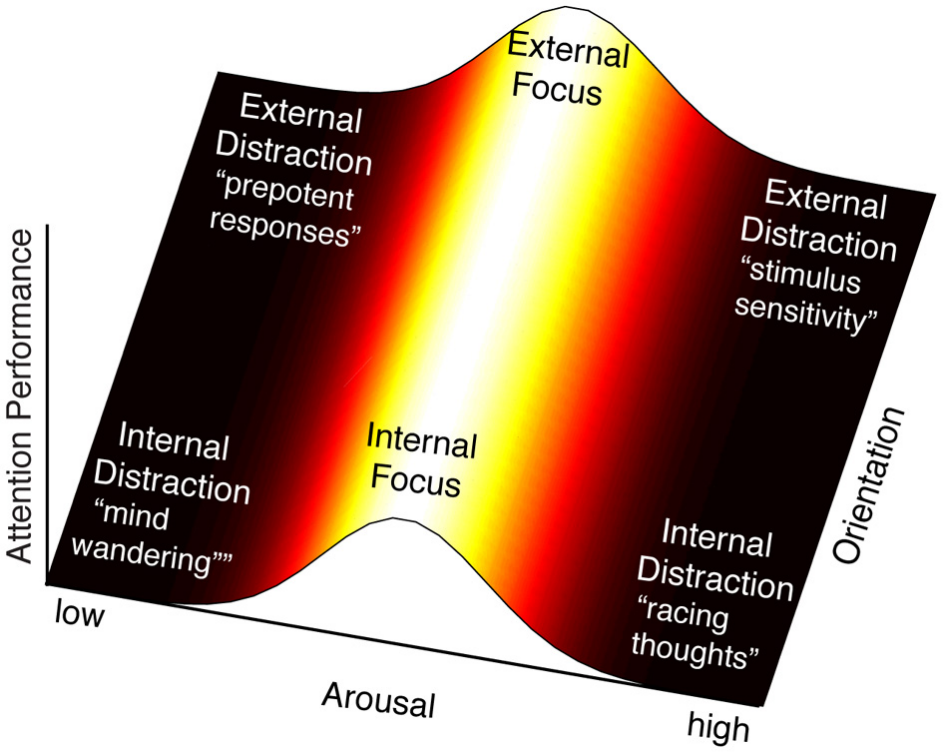
**By intersecting arousal and input orientation, we observe six states of attention control: focus directed internally (e.g., problem solving) or externally (e.g., reading a book), and four classes of deficits of attention control.** The region of maximal attention performance lies along the length of the orientation axis, where it intersects the midpoint of the arousal axis. At this point is the peak of the proposed arousal-attention function, described by the Yerkes-Dodson curve, when attention control efficacy is maximal. The proposed effect of LC-NE on FPN neural response is thought to result in a sensitized response when arousal is high and a sluggish response when arousal is low. The interpretation of these extremes in terms of attention deficits varies with the orientation of attention control. High arousal is interpreted as over-activity of the FPN, which could produce racing thoughts or rumination when attention is oriented internally (bottom-right), and stimulus sensitivity when attention is oriented externally (top-right). Low arousal is interpreted as under-activity of the FPN, which would result in automated, “bottom-up,” responses. For internally oriented attention this may be analogous to mind wandering (bottom-left). For externally oriented attention (top-left) this may be analogous to attention responses that are based on prepotency of stimuli (e.g., tendency to read words rather than name ink color in the Stroop task) or salience (e.g., attention capture).

The notion that multiple influences can modify the behavior of the FPN implies that attention control can take on multiple states that are determined by the context of its inputs and systemic influences, or more simply, by its system interactions. For instance, if we take the above examples of attention control inputs (internal/external) and activation (low/high arousal) and explore the product of their interactions along two axes, a landscape of attention control *states* emerges (Figure 2). Following the orientation axis, we see that attention control can be oriented internally or externally. We show no variability in orientation, acknowledging that it is categorical. Following the horizontal arousal axis, we see that attention control efficacy varies with arousal level with an optimum in the middle—reflecting the Yerkes-Dodson relationship exemplified by this system (Aston-Jones et al., 1999). Hence most efficacious attention states occur at the peak of this function, though the orientation can vary (e.g., focus can be directed internally such as when problem solving, or externally such as when reading a book), whereas in the extremes of the arousal axis, attention lapses occur. Scanning the resulting landscape, two important observations arise.

The first is that attention lapses can take on multiple flavors. In this analysis we observe four domains, produced by crossing arousal states with orientation of attention. If arousal is high, we predict that FPN will be excessively responsive to all stimuli and will therefore fail in discriminating between relevant and irrelevant inputs. If attention were oriented externally, this may be manifest as oversensitivity to external stimuli, whereas if attention were oriented internally, it might translate into racing thoughts (perhaps rumination). If however arousal is low, we predict that the FPN response will be sluggish, resulting in reduced responsivity to stimuli. Again discrimination between relevant and irrelevant stimuli would be compromised. In this case, if attention were oriented externally, we would expect behaviors to be driven by the most salient or most automatic responses (“bottom-up”) since minimal control is applied to inputs. Similarly if attention were oriented internally, we would expect the presence of mind wandering, in which internal cognition drifts from one topic to another. This is also the state in which externally oriented attention would be vulnerable to drifting to internal content and, similarly, internal orientation could drift to external content. Note that in all four states the outward symptom would be poor attention to the task at hand, but for very different reasons.

This perspective raises some questions regarding attention control mechanisms, especially with regard to internal cognition. The present synthesis implies that internal cognition is subject to the same rules of attention control that apply to external inputs: enhancement of relevant information and suppression of irrelevant information. Accordingly, distractions *within* internal cognition ought to be manifest much like those for external signals. As an example, consider the case where you attempt to meditate, but instead drift into thinking about work. Or imagine that you are trying to retrieve the name of a high-school friend (e.g., “Jenny”), but your memory keeps drifting to your colleague who has similar sounding last name (e.g., “Jensen”). In both cases, a potent unrelated thought captures your attention in the internal modality—much like when a loud sound captures your attention in the external modality. Therefore *relevant* and *irrelevant* signals may be defined for internal cognition much like external signals such as sights and sounds, and a correct response of the system would be for the FPN to suppress those irrelevant work thoughts, or the competing memory.

What does it mean for FPN to suppress an internal thought? Can thoughts be conceived as isomorphic with levels of cortical activity, much like sounds and sights are isomorphic with levels of activity in auditory and visual cortices? If so, what are these cortical regions or networks that would be modulated? Would the structure of internal thought representations require an additional control system that interacts with the FPN? More generally, how would we measure lapses that occur *within* the internal modality? While certain aspects of attention control may be preserved between internal and external modalities, it is possible that asymmetries exist. Furthermore, while we describe a landscape of attentional states, we have not addressed how transitions occur between these states. How do the observed fluctuations in network activity and in behavioral performance relate to transitions in attentional states? For example, when FPN activity is low does this create a lower barrier for attention to wander or be captured? These are important questions that beg further investigation.

Our second, and related, observation is that current models of attention control are based largely on the study of externally oriented attention. Accordingly, investigations of attention control impairments are restricted largely to the upper half of Figure 2, more precisely to the upper left. Interestingly, while distractibility has been ascribed to a failure of the attention control network, the cause is not transparent. While it is certainly likely that in some disorders the FPN is impaired, in other instances an apparent dysfunction of FPN may be accounted for by low arousal decreasing activation of a normally functioning FPN. The implication of this proposition is that apparent dysfunctions of FPN may arise through multiple mechanisms. An interesting test case in this regard is ADHD. Key symptoms of this disorder have been impairments of working memory and response inhibition (Barkley, 1997; Tannock, 1998), leading to the inference that prefrontal cortex function, which subsumes core nodes of the FPN, is dysfunctional (Castellanos and Tannock, 2002; Arnsten, 2006; Casey and Riddle, 2012). Yet, the disorder also has been associated with an impairment of arousal, possibly due to an underlying noradrenergic disorder (van der Meere and Sergeant, 1988; McCracken, 1991; Biederman and Spencer, 1999).

Several questions arises: are the apparent attention control symptoms mediated, at least in part, by an underlying deficit in arousal and, therefore, the sustaining or engaging of attention control (Huang-Pollock and Nigg, 2003; Huang-Pollock et al., 2005; Castellanos et al., 2006; Friedman-Hill et al., 2010)? Are the fluctuations in attention control within an individual related to interactions of FPN with the LC-NE system? For instance, the fluctuations of attention control observed by Castellanos et al. (2005), more pronounced in children with ADHD, had a period of approximately 15 s. Is this frequency correlated with the fluctuations of the LC-NE system in this group? Is the amplification of these fluctuations related to an aberration in the cellular properties of the neuromodulatory projections?

## Conclusion

Our objective in this perspective is to highlight the significance of known fluctuations of attention control. We suggest that sources of these fluctuations consist of two categories, systemic and input, and that may they be thought of as interactions between FPN and other neural systems. We have presented a possible landscape of attention control that may result from the interactions of these systems. Recognizing that this demonstration is incomplete—inevitably other neuromodulators, other networks and associated system interactions are involved—we present this short perspective to highlight the increasing emphasis on and exciting research that has emerged in describing the brain in terms of network interactions. We believe that understanding of these interactions in the context of attention control fluctuations is imminent and will lead to an improved characterization of the dynamics of attention control and of its impairments.

### Conflict of interest statement

The authors declare that the research was conducted in the absence of any commercial or financial relationships that could be construed as a potential conflict of interest.

## References

[B1] Andrews-Hanna J. R. (2012). The brain's default network and its adaptive role in internal mentation. Neuroscientist 18, 251–270 10.1177/1073858411403316 21677128 PMC3553600

[B2] Andrews-Hanna J. R. Reidler J. S. Huang C. Buckner R. L. (2010). Evidence for the default network's role in spontaneous cognition. J. Neurophysiol. 104, 322–335 10.1152/jn.00830.2009 20463201 PMC2904225

[B3] Arnsten A. F. (2006). Fundamentals of attention-deficit/hyperactivity disorder: circuits and pathways. J. Clin. Psychiatry 67 Suppl 8, 7–12 16961424

[B4] Aston-Jones G. Bloom F. E. (1981). Activity of norepinephrine-containing locus coeruleus neurons in behaving rats anticipates fluctuations in the sleep-waking cycle. J. Neurosci. 1, 876–886 7346592 10.1523/JNEUROSCI.01-08-00876.1981 PMC6564235

[B5] Aston-Jones G. Cohen J. D. (2005). An integrative theory of locus coeruleus-norepinephrine function: adaptive gain and optimal performance. Annu. Rev. Neurosci. 28, 403–450 10.1146/annurev.neuro.28.061604.135709 16022602

[B6] Aston-Jones G. Rajkowski J. Cohen J. (1999). Role of locus coeruleus in attention and behavioral flexibility. Biol. Psychiatry 46, 1309–1320 10.1016/S0006-3223(99)00140-7 10560036

[B7] Barkley R. A. (1997). Behavioral inhibition, sustained attention, and executive functions: constructing a unifying theory of ADHD. Psychol. Bull. 121, 65–94 10.1037/0033-2909.121.1.65 9000892

[B8] Barron E. Riby L. M. Greer J. Smallwood J. (2011). Absorbed in thought: the effect of mind wandering on the processing of relevant and irrelevant events. Psychol. Sci. 22, 596–601 10.1177/0956797611404083 21460338

[B9] Berridge C. W. Waterhouse B. D. (2003). The locus coeruleus-noradrenergic system: modulation of behavioral state and state-dependent cognitive processes. Brain Res. Brain Res. Rev. 42, 33–84 10.1016/S0165-0173(03)00143-7 12668290

[B10] Biederman J. Spencer T. (1999). Attention-deficit/hyperactivity disorder (ADHD) as a noradrenergic disorder. Biol. Psychiatry 46, 1234–1242 10.1016/S0006-3223(99)00192-4 10560028

[B11] Binder J. R. Frost J. A. Hammeke T. A. Bellgowan P. S. F. Rao S. M. Cox R. W. (1999). Conceptual processing during the conscious resting state: a functional MRI study. J. Cogn. Neurosci. 11, 80–93 10.1162/089892999563265 9950716

[B12] Botvinick M. M. Braver T. S. Barch D. M. Carter C. S. Cohen J. D. (2001). Conflict monitoring and cognitive control. Psychol. Rev. 108, 624–652 10.1037/0033-295X.108.3.624 11488380

[B13] Braver T. S. Cohen J. D. (2000). On the control of control: the role of dopamine in regulating prefrontal function and working memory, in Attention and Performance XVIII, eds Monsell S. Driver J. (Cambridge, MA: MIT Press), 713–737

[B14] Briand L. A. Gritton H. Howe W. M. Young D. A. Sarter M. (2007). Modulators in concert for cognition: modulator interactions in the prefrontal cortex. Prog. Neurobiol. 83, 69–91 10.1016/j.pneurobio.2007.06.007 17681661 PMC2080765

[B15] Buckner R. L. Andrews-Hanna J. R. Schacter D. L. (2008). The brain's default network - Anatomy, function, and relevance to disease. Ann. N.Y. Acad. Sci. 1124, 1–38 18400922 10.1196/annals.1440.011

[B16] Capotosto P. Babiloni C. Romani G. L. Corbetta M. (2009). Frontoparietal cortex controls spatial attention through modulation of anticipatory alpha rhythms. J. Neurosci. 29, 5863–5872 10.1523/JNEUROSCI.0539-09.2009 19420253 PMC2692025

[B17] Casey B. J. Riddle M. (2012). Typical and atypical development of attention, in Cognitive Neuroscience of Attention, 2nd Edn., ed Posner M. (New York, NY: Guilford Press), 514

[B18] Castellanos F. X. Sonuga-Barke E. J. S. Milham M. P. Tannock R. (2006). Characterizing cognition in ADHD: beyond executive dysfunction. Trends Cogn. Sci. 10, 117–123 10.1016/j.tics.2006.01.011 16460990

[B19] Castellanos F. X. Sonuga-Barke E. J. S. Scheres A. Di Martino A. Hyde C. Walters J. R. (2005). Varieties of attention-deficit/hyperactivity disorder-related intra-individual variability. Biol. Psychiatry 57, 1416–1423 10.1016/j.biopsych.2004.12.005 15950016 PMC1236991

[B20] Castellanos F. X. Tannock R. (2002). Neuroscience of attention-deficit/hyperactivity disorder: the search for endophenotypes. Nat. Rev. Neurosci. 3, 617–628 12154363 10.1038/nrn896

[B21] Christoff K. (2012). Undirected thought: neural determinants and correlates. Brain Res. 1428, 51–59 10.1016/j.brainres.2011.09.060 22071565

[B22] Christoff K. Gordon A. M. Smallwood J. Smith R. Schooler J. W. (2009). Experience sampling during fMRI reveals default network and executive system contributions to mind wandering. Proc. Natl. Acad. Sci. U.S.A. 106, 8719–8724 10.1073/pnas.0900234106 19433790 PMC2689035

[B23] Corbetta M. Patel G. Shulman G. L. (2008). The reorienting system of the human brain: from environment to theory of mind. Neuron 58, 306–324 10.1016/j.neuron.2008.04.017 18466742 PMC2441869

[B24] Corbetta M. Shulman G. L. (2002). Control of goal-directed and stimulus-driven attention in the brain. Nat. Rev. Neurosci. 3, 201–215 10.1038/nrn755 11994752

[B25] Coull J. T. (1998). Neural correlates of attention and arousal: insights from electrophysiology, functional neuroimaging and psychopharmacology. Prog. Neurobiol. 55, 343–361 10.1016/S0301-0082(98)00011-2 9654384

[B26] Desimone R. Duncan J. (1995). Neural mechanisms of selective visual-attention. Annu. Rev. Neurosci. 18, 193–222 10.1146/annurev.ne.18.030195.001205 7605061

[B27] Dosenbach N. U. F. Visscher K. M. Palmer E. D. Miezin F. M. Wenger K. K. Kang H. S. C. (2006). A core system for the implementation of task sets. Neuron 50, 799–812 10.1016/j.neuron.2006.04.031 16731517 PMC3621133

[B28] Foote S. L. Bloom F. E. Aston-Jones G. (1983). Nucleus locus ceruleus–new evidence of anatomical and physiological specificity. Physiol. Rev. 63, 844–914 6308694 10.1152/physrev.1983.63.3.844

[B29] Foote S. L. Freedman R. Oliver A. P. (1975). Effects of putative neurotransmitters on neuronal-activity in monkey auditory-cortex. Brain Res. 86, 229–242 10.1016/0006-8993(75)90699-X 234774

[B30] Foote S. L. Morrison J. H. (1987). Extrathalamic modulation of cortical function. Annu. Rev. Neurosci. 10, 67–95 10.1146/annurev.ne.10.030187.000435 3551766

[B31] Fox M. D. Corbetta M. Snyder A. Z. Vincent J. L. Raichle M. E. (2006). Spontaneous neuronal activity distinguishes human dorsal and ventral attention systems. Proc. Natl. Acad. Sci. U.S.A. 103, 10046–10051 10.1073/pnas.0604187103 16788060 PMC1480402

[B32] Fox M. D. Raichle M. E. (2007). Spontaneous fluctuations in brain activity observed with functional magnetic resonance imaging. Nat. Rev. Neurosci. 8, 700–711 10.1038/nrn2201 17704812

[B33] Fox M. D. Snyder A. Z. Vincent J. L. Corbetta M. Van Essen D. C. Raichle M. E. (2005). The human brain is intrinsically organized into dynamic, anticorrelated functional networks. Proc. Natl. Acad. Sci. U.S.A. 102, 9673–9678 10.1073/pnas.0504136102 15976020 PMC1157105

[B34] Friedman-Hill S. R. Wagman M. R. Gex S. E. Pine D. S. Leibenluft E. Ungerleider L. G. (2010). What does distractibility in ADHD reveal about mechanisms for top-down attentional control? Cognition 115, 93–103 10.1016/j.cognition.2009.11.013 20096409 PMC2830348

[B35] Gazzaley A. Rissman J. Cooney J. Rutman A. Seibert T. Clapp W. (2007). Functional interactions between prefrontal and visual association cortex contribute to top-down modulation of visual processing. Cereb. Cortex 17, I125–I135 10.1093/cercor/bhm113 17725995 PMC4530799

[B36] Giambra L. M. (1995). A laboratory method for investigating influences on switching attention to task-unrelated imagery and thought. Conscious. Cogn. 4, 1–21 10.1006/ccog.1995.1001 7497092

[B37] Gordon A. Smith R. Keramatian K. Luus B. Weinberg A. Smallwood J. (2007). Mind-wandering, awareness, and task performance: an fMRI study. Can. J. Exp. Psychol. 61, 366 19433790

[B38] Gusnard D. A. Akbudak E. Shulman G. L. Raichle M. E. (2001). Medial prefrontal cortex and self-referential mental activity: relation to a default mode of brain function. Proc. Natl. Acad. Sci. U.S.A. 98, 4259–4264 10.1073/pnas.071043098 11259662 PMC31213

[B39] Huang-Pollock C. L. Nigg J. T. (2003). Searching for the attention deficit in attention deficit hyperactivity disorder: the case of visuospatial orienting. Clin. Psychol. Rev. 23, 801–830 10.1016/S0272-7358(03)00073-4 14529699

[B40] Huang-Pollock C. L. Nigg J. T. Carr T. H. (2005). Deficient attention is hard to find: applying the perceptual load model of selective attention to attention deficit hyperactivity disorder subtypes. J. Child Psychol. Psychiatry 46, 1211–1218 10.1111/j.1469-7610.2005.00410.x 16238668

[B41] Johnson S. C. Baxter L. C. Wilder L. S. Pipe J. G. Heiserman J. E. Prigatano G. P. (2002). Neural correlates of self-reflection. Brain 125, 1808–1814 10.1093/brain/awf181 12135971

[B42] Kam J. W. Y. Dao E. Farley J. Fitzpatrick K. Smallwood J. Schooler J. W. (2011). Slow fluctuations in attentional control of sensory cortex. J. Cogn. Neurosci. 23, 460–470 10.1162/jocn.2010.21443 20146593

[B43] Leech R. Braga R. Sharp D. J. (2012). Echoes of the brain within the posterior cingulate cortex. J. Neurosci. 32, 215–222 10.1523/JNEUROSCI.3689-11.2012 22219283 PMC6621313

[B44] Mason M. F. Norton M. I. Van Horn J. D. Wegner D. M. Grafton S. T. Macrae C. N. (2007). Wandering minds: the default network and stimulus-independent thought. Science 315, 393–395 10.1126/science.1131295 17234951 PMC1821121

[B45] Mason S. T. Iversen S. D. (1978). Reward, attention and dorsal noradrenergic bundle. Brain Res. 150, 135–148 10.1016/0006-8993(78)90658-3 667617

[B46] Mazoyer B. Zago L. Mellet E. Bricogne S. Etard O. Houde O. (2001). Cortical networks for working memory and executive functions sustain the conscious resting state in man. Brain Res. Bull. 54, 287–298 10.1016/S0361-9230(00)00437-8 11287133

[B47] McCracken J. T. (1991). A two-part model of stimulant action on attention-deficit hyperactivity disorder in children. J. Neuropsychiatry Clin. Neurosci. 3, 201–209 1687963 10.1176/jnp.3.2.201

[B48] McVay J. C. Kane M. J. (2009). Conducting the train of thought: working memory capacity, goal neglect, and mind wandering in an executive-control task. J. Exp. Psychol. Learn. Mem. Cogn. 35, 196–204 10.1037/a0014104 19210090 PMC2750806

[B49] McVay J. C. Kane M. J. (2010). Does mind wandering reflect executive function or executive failure? Comment on Smallwood and Schooler (2006) and Watkins (2008). Psychol. Bull. 136, 188–197 10.1037/a0018298 20192557 PMC2850105

[B50] Miller E. K. Cohen J. D. (2001). An integrative theory of prefrontal cortex function. Annu. Rev. Neurosci. 24, 167–202 10.1146/annurev.neuro.24.1.167 11283309

[B51] Monto S. Palva S. Voipio J. Palva J. M. (2008). Very slow EEG fluctuations predict the dynamics of stimulus detection and oscillation amplitudes in humans. J. Neurosci. 28, 8268–8272 10.1523/JNEUROSCI.1910-08.2008 18701689 PMC6670577

[B52] Moore R. Y. Bloom F. E. (1979). Central catecholamine neuron systems–anatomy and physiology of the norepinephrine and epinephrine systems. Annu. Rev. Neurosci. 2, 113–168 10.1146/annurev.ne.02.030179.000553 231924

[B53] Moruzzi G. Magoun H. W. (1949). Brain stem reticular formation and activation of EEG. Electroencephalogr. Clin. Neurophysiol. 1, 455–473 10.1016/0013-4694(49)90219-9 18421835

[B54] Posner M. I. Dehane S. (1994). Attentional networks. Trends Neural Sci. 17, 75–79 10.1016/0166-2236(94)90078-7 7512772

[B55] Posner M. I. Petersen S. E. (1990). The attention system of the human brain. Annu. Rev. Neurosci. 13, 25–42 10.1146/annurev.ne.13.030190.000325 2183676

[B56] Raichle M. E. (2011). The restless brain. Brain Connect. 1, 3–12 10.1089/brain.2011.0019 22432951 PMC3621343

[B57] Raichle M. E. Macleod A. M. Snyder A. Z. Powers W. J. Gusnard D. A. Shulman G. L. (2001). A default mode of brain function. Proc. Natl. Acad. Sci. U.S.A. 98, 676–682 10.1073/pnas.98.2.676 11209064 PMC14647

[B58] Rokem A. Landau A. N. Garg D. Prinzmetal W. Silver M. A. (2010). Cholinergic enhancement increases the effects of voluntary attention but does not affect involuntary attention. Neuropsychopharmacology 35, 2538–2544 10.1038/npp.2010.118 20811340 PMC2978769

[B59] Roussel B. Buguet A. Bobillier P. Jouvet M. (1967). [Locus ceruleus, paradoxal sleep, and cerebral noradrenaline]. C. R. Seances Soc. Biol. Fil. 161, 2537–2541 4302168

[B60] Ruff C. C. Driver J. (2006). Attentional preparation for a lateralized visual distractor: behavioral and fMRI evidence. J. Cogn. Neurosci. 18, 522–538 10.1162/jocn.2006.18.4.522 16768358

[B61] Schooler J. W. Smallwood J. Christoff K. Handy T. C. Reichle E. D. Sayette M. A. (2011). Meta-awareness, perceptual decoupling and the wandering mind. Trends Cogn. Sci. 15, 319–326 21684189 10.1016/j.tics.2011.05.006

[B62] Shulman G. L. Fiez J. A. Corbetta M. Buckner R. L. Miezin F. M. Raichle M. E. (1997). Common blood flow changes across visual tasks.2. Decreases in cerebral cortex. J. Cogn. Neurosci. 9, 648–663 10.1162/jocn.1997.9.5.648 23965122

[B63] Smallwood J. (2012). Understanding unconstrained mental processes during waking thought: a cognitive neuroscience exploration of the wandering mind. Can. J. Exp. Psychol. 66, 316

[B64] Smallwood J. Beach E. Schooler J. W. Handy T. C. (2008). Going AWOL in the brain: mind wandering reduces cortical analysis of external events. J. Cogn. Neurosci. 20, 458–469 10.1162/jocn.2008.20037 18004943

[B65] Smallwood J. Brown K. Baird B. Schooler J. W. (2012). Cooperation between the default mode network and the frontal-parietal network in the production of an internal train of thought. Brain Res. 1428, 60–70 10.1016/j.brainres.2011.03.072 21466793

[B66] Smallwood J. McSpadden M. Schooler J. W. (2007). The lights are on but no one's home: meta-awareness and the decoupling of attention when the mind wanders. Psychonom. Bull. Rev. 14, 527–533 10.3758/BF03194102 17874601

[B67] Smallwood J. Schooler J. W. (2006). The restless mind. Psychol. Bull. 132, 946–958 10.1037/0033-2909.132.6.946 17073528

[B68] Spreng R. N. Sepulcre J. Turner G. R. Stevens W. D. Schacter D. L. (2013). Intrinsic architecture underlying the relations among the default, dorsal attention, and frontoparietal control networks of the human brain. J. Cogn. Neurosci. 25, 74–86 10.1162/jocn_a_00281 22905821 PMC3816715

[B69] Spreng R. N. Stevens W. D. Chamberlain J. P. Gilmore A. W. Schacter D. L. (2010). Default network activity, coupled with the frontoparietal control network, supports goal-directed cognition. Neuroimage 53, 303–317 10.1016/j.neuroimage.2010.06.016 20600998 PMC2914129

[B70] Sridharan D. Levitin D. J. Menon V. (2008). A critical role for the right fronto-insular cortex in switching between central-executive and default-mode networks. Proc. Natl. Acad. Sci. U.S.A. 105, 12569–12574 10.1073/pnas.0800005105 18723676 PMC2527952

[B71] Stawarczyk D. Majerus S. Maquet P. D'argembeau A. (2011). Neural correlates of ongoing conscious experience: both task-unrelatedness and stimulus-independence are related to default network activity. PLoS ONE 6:e16997 10.1371/journal.pone.0016997 21347270 PMC3038939

[B72] Tannock R. (1998). Attention deficit hyperactivity disorder: advances in cognitive, neurobiological, and genetic research. J. Child Psychol. Psychiatry 39, 65–99 10.1111/1469-7610.00304 9534087

[B73] van der Meere J. Sergeant J. (1988). Focused attention in pervasively hyperactive children. J. Abnorm. Child Psychol. 16, 627–639 10.1007/BF00913474 3216072

[B74] Vincent J. L. Kahn I. Snyder A. Z. Raichle M. E. Buckner R. L. (2008). Evidence for a frontoparietal control system revealed by intrinsic functional connectivity. J. Neurophysiol. 100, 3328–3342 10.1152/jn.90355.2008 18799601 PMC2604839

[B75] Weissman D. H. Roberts K. C. Visscher K. M. Woldorff M. G. (2006). The neural bases of momentary lapses in attention. Nat. Neurosci. 9, 971–978 10.1038/nn1727 16767087

[B76] Weissman D. H. Warner L. M. Woldorff M. G. (2009). Momentary reductions of attention permit greater processing of irrelevant stimuli. Neuroimage 48, 609–615 10.1016/j.neuroimage.2009.06.081 19596451 PMC2738758

